# Serious Hemorrhagic Complications After Successful Treatment of Hematopoietic Stem Cell Transplantation-Associated Thrombotic Microangiopathy With Defibrotide in Pediatric Patient With Myelodysplastic Syndrome

**DOI:** 10.3389/fped.2020.00155

**Published:** 2020-05-05

**Authors:** Alexandra Laberko, Marina Aksenova, Irina Shipitsina, Igor Khamin, Anna Shcherbina, Dmitry Balashov, Alexei Maschan

**Affiliations:** ^1^Immunology, Dmitry Rogachev National Medical Center of Pediatric Hematology, Oncology and Immunology, Moscow, Russia; ^2^Nephrology, Dmitry Rogachev National Medical Center of Pediatric Hematology, Oncology and Immunology, Moscow, Russia; ^3^Nephrology, Y. Veltischev Research and Clinical Institute for Pediatrics at N. Pirogov Russian National Research Medical University, Moscow, Russia; ^4^Hematopoietic Stem Cell Transplantation, Dmitry Rogachev National Medical Center of Pediatric Hematology, Oncology and Immunology, Moscow, Russia; ^5^Intensive Care, Dmitry Rogachev National Medical Center of Pediatric Hematology, Oncology and Immunology, Moscow, Russia

**Keywords:** hematopoietic stem cell transplantation, myelodysplastic syndrome, thrombotic microangiopathy, defibrotide, renal bleeding

## Abstract

**Background:** Transplant-associated thrombotic microangiopathy (TAM) is a life-threatening complication of hematopoietic stem cell transplantation (HSCT). There is some evidence of endothelial injury playing a significant role in TAM development. The efficacy of defibrotide was demonstrated for prophylaxis and treatment of another HSCT-associated endothelial damage syndrome—liver veno-occlusive disease. The data for defibrotide usage in TAM are limited.

**Case Description:** A 9-year old boy underwent HSCT from a matched unrelated donor for monosomy seven-associated myelodysplastic syndrome treatment. A myeloablative preparative regimen and post-transplant immunosuppression with cyclophosphamide on days +3 and +4 and a combination of tacrolimus with mycophenolate mofetil from day +5 were used. From day +61, sustained fever with progressive neurologic impairment and no evidence of infection was observed. On day +68, the patient developed severe TAM with acute kidney injury requiring renal replacement therapy (RRT). Defibrotide therapy 25 mg/kg/day was administered for 7 days with resolution of TAM symptoms. It was followed by multiple hemorrhagic episodes—epistaxis, hemorrhagic cystitis, and renal hemorrhage, which are presumed to be the complications of defibrotide therapy.

**Conclusion:** Defibrotide could be an effective therapy for TAM, but adequate doses, duration of therapy, and drug safety profile both for pediatric and adult patients need to be evaluated by randomized prospective studies.

## Background

Thrombotic microangiopathy is a rare complication of hematopoietic stem cell transplantation (HSCT). The most characteristic features are microangiopathic hemolytic anemia and thrombocytopenia, accompanied by a variable degree of acute or subacute kidney injury (AKI) and central nervous system (CNS) involvement (1). Transplant-associated thrombotic microangiopathy (TAM) is one of the syndromes associated with endothelial damage, which are usually caused by a complex exposure of chemo/radiotherapy, transplantation-associated infectious processes, alloimmune reactions, and others (2). One of the most clearly defined causative factors for TAM development is the use of calcineurin inhibitors (3, 4).

The approaches to TAM treatment are poorly established, and TAM-associated mortality remains high (5, 6). Defibrotide is a polydeoxyribonucleotide-based drug, which is currently commonly used for prophylaxis and treatment of another life-threatening HSCT-associated endothelial damage syndrome—liver veno-occlusive disease (VOD) (7, 8). The mechanism of defibrotide therapeutic action remains unknown, but there are a few reports of its efficacy in other endothelial injury syndromes, including TAM (9, 10), and one report of its efficacy as monotherapy of TAM in pediatric patients (11).

Here, we present our experience of defibrotide administration in a pediatric patient with TAM.

## Case Presentation

A male patient was diagnosed with moderate transient neutropenia of infancy (absolute neutrophil count below 1,000/mm^3^, but above 500/mm^3^). By 4 years of age, he developed recurrent respiratory infections. Immunologic investigation showed normal lymphocyte subsets, serum immunoglobulins, and phagocyte function. The patient demonstrated facial dysmorphism and moderate cognitive difficulties, and from 6 years of age developed recurrent warts. At the age of 7 years, after an episode of infectious mononucleosis, the patient presented with thrombocytopenia (platelet count 50 × 10^9^/L) and exacerbation of neutropenia with transient decrease of neutrophils <0.5 × 10^9^/L. At the age of 8 years, BM investigation revealed multilineage dysplasia with no blast excess and monosomy 7 [fluorescent *in situ* hybridization (FISH); Vysis LSI D7S486/CEP7; Abbott Laboratories, Edmonds, WA, USA] in 50% of cells; therefore, monosomy seven-associated myelodysplastic syndrome (MDS): refractory cytopenia of childhood was diagnosed (12). No mutations in WAS, GATA2 (Sanger sequencing method), or deletion 22q11.2 (FISH; Vysis LSI D7S486/CEP7; Abbott Laboratories; Edmonds, WA, USA) were detected. A diepoxybutane test revealed no increase in chromosome breakage.

At the age of 9 years, bone marrow transplantation from an HLA 10/10 matched unrelated donor was performed. The conditioning regimen included total doses of treosulfan−42 g/m^2^, fludarabin−150 mg/m^2^, and thiotepa−300 mg/m^2^. The doses of graft cells were nucleated cells−3.88 × 10^8^/kg, CD34^+^ 2.8 × 10^6^/kg, CD3_+_ 0.4 × 10^8^/kg. Graft-versus-host disease (GVHD) prophylaxis consisted of cyclophosphamide 50 mg/kg delivered on day +3 and +4 after HSCT, followed by tacrolimus and mycophenolate mofetil, 30 mg/kg from day +5. Neutrophil and platelet engraftment occurred by days +21 and +28, respectively.

At an early post-transplant period, the patient developed arterial hypertension, which was controlled with two hypotensive drugs. By day +37, creatinine had increased to 80 μmol/L, but which completely resolved with a short pause in tacrolimus therapy. Blood concentration of tacrolimus day +35 was 7.3 ng/mL, and haptoglobin and schistocytes were within the normal ranges. After 10 days, tacrolimus therapy was resumed at a lower dose.

On day +58, the patient developed persistent fever, resistant to antibiotics. Cytomegalovirus (CMV) was detected (real-time PCR) in blood (2,730 copies/mL) and bronchoalveolar lavage (71,000 copies/mL) with no signs of pneumonia on thoracic computerized tomography (CT). CMV infection resolved after ganciclovir treatment. On day +61, the patient developed somnolence for 4 days, followed by tremor of the upper limbs and inferior jaw, and insomnia with no hypertension. Cranial magnetic resonance investigation (MRI) was normal. Tacrolimus therapy was discontinued. In 2 days (day +68), tremor and insomnia resolved, but the patient again became somnolent and developed acute kidney failure with anuria, mild hypertension (maximum 130/85 mmHg), elevation of lactate dehydrogenase, decrease of platelet and hemoglobin levels (the blood and urine test results are shown in Table 1), and was admitted to intensive care unit for renal replacement therapy (RRT) (PRISMAFLEX System (Baxter International, USA). The patient's symptoms were consistent with a diagnosis of TAM, and so defibrotide therapy 25 mg/kg/day was started. On the second day of defibrotide therapy, resolution of fever and somnolence was observed. At day 4, the patient developed epistaxis, followed by macrohematuria. Coagulation tests were constantly normal. Ultrasound, MRI, and CT investigations revealed bilateral renal subcapsular hematomas (71 × 23 × 90 mm and 57 × 18 × 93 mm), with extension into paranephric fat. Owing to renal function improvement, RRT and defibrotide therapy were discontinued on the 7th day of treatment. Three days later, the patient developed hemorrhagic cystitis (with lower abdominal pain, ultrasound thickening of bladder wall, and intrabladder clotting), negative for BK, CMV, and adenovirus in urine. Hemorrhagic cystitis was managed with no aggressive intervention, and support with platelets and red blood cells (platelet level sustained above 75 × 10 ^*^ 9/L). Renal bleeding and cystitis resolved spontaneously within 7 days after commencing. Diuresis normalized within 3 weeks of anuria onset, with reduction and stabilization of creatinine and urea levels within 2 months.

**Table 1:** Monitoring of clinical and laboratory in patients with TAM.

**Parameter**	**Day +61**	**Day +68**	**Day +75**	**Day +120**	Normal ranges^*^
Clinical symptoms	-Neurologic impairment -Fever	-Anuria -Neurologic impairment -Moderate arterial hypertension -Fever	-Bleeding -Severe arterial hypertension	-Moderate arterial hypertension	
Treatment	Tacrolimus	RRT and defibrotide initiation	RRT and defibrotide stop		
**Blood tests**					
Creatinine, μmol/L	50.9	472	150	75.5	<56
Urea, mmol/L	3.4	19	10	3.7	2.5–6
Lactate dehydrogenase ME/L	388	577	703	305	<332
Haptoglobin, g/L	0.14	0.44		0.05	0.3–2
Hemoglobin, g/L	106	71	105^**^	92^**^	115–138
Platelets, × 10^9^/L	152	104	75^**^	133	150–400
Shistocytes, %_0_	0	2		2	<2.7
Coombs test		Negative		Negative	
Tacrolimus, ng/mL	5.9	0.5			
**Coagulation tests**					
Fibrinogen, g/L		3.06	4.66		
Prothrombin time, %		89	87		70–120
Activated partial thromboplastin time, s		30.1	12.9		25.1–36.5
Thrombin time, s		23.7	24.3		15.8–24.9
C3, g/L			0.74		0.66–1.1
**Urinalysis**					
Urine total protein, g/L	1	5		0.2	<0.5

* *Our laboratory normal value for age*.

** *With transfusion support*.

Currently, 1 year after HSCT, the patient has full donor chimerism, features of immune recovery, and no signs of MDS. Renal investigation revealed chronic kidney disease (CKD), stage 2 (13) with estimated glomerular filtration rate of 63 mL/min/1.73 m^2^. Owing to persistent proteinuria (0.1–0.5 g/L), treatment with enalapril (0.1 mg/kg/day) was started.

Whole exome sequencing (NextSeq500 platform; Illumina, CA, USA) performed with patient's stored pre-transplant blood sample revealed no pre-transplant disease causing gene mutations.

## Discussion

TAM is an HSCT-associated endothelial damage syndrome. The most common TAM presentation is thrombocytopenia and microangiopathic hemolytic anemia with ischemic patterns of organ damage, related to intravascular platelet aggregation and microthrombi formation (14). Micro-vascular occlusion predominantly causes kidney and neurologic impairment, yet there is some evidence of impairment of other organs, such as the lungs or gastrointestinal tract (1). Unfortunately, clinical features of TAM are non-specific and can overlap or mimic GVHD, infection, or drug toxicity. Therefore, diagnostic TAM criteria have changed dramatically over the past decade (15). Most TAM criteria propose evidence of erythrocyte fragmentation (shistocytosis and haptoglobin decrease), which was not observed at the time of TAM development in our patient. Recently, Jodele et al. (5) have defined more accurate criteria with early (LDH elevation, proteinuria, and hypertension) and late (*de novo* anemia and thrombocytopenia, shistocytosis, and terminal complement cascade activation) TAM symptoms. Interestingly, in our patient we observed fulminant TAM development with preceding CNS impairment, followed by simultaneous presentation of acute kidney failure and both early and late TAM clinical and laboratory symptoms. Although, ADAMTS13 was not measured to exclude thrombotic thrombocytopenic purpura (TTP) (16), we believe that TTP could not resolve so promptly with no specific pathogenetic therapy.

TAM in our patient was likely caused by tacrolimus toxicity (6, 17), which was not dose dependent (the estimated concentration of tacrolimus in blood in both episodes of tacrolimus toxicity was within the therapeutic range). We also presume the possible role of the patient's underlying disease to increase cell sensitivity to toxic agent exposure. Owing to the combination of features of immunologic (warts), hematologic (early-onset intermittent neutropenia evolving to MDS associated with monosomy 7), and cognitive impairments in combination with facial dysmorphism, the patient is suspected to have one of the DNA-repair disorders (18–20). However, whole exome sequencing did not reveal any disease causative gene mutations.

There are no reliable, and only a few, potential strategies for TAM treatment. One possible curative option is complement cascade inhibition with eculizumab (21, 22) or, more recently, narsoplimab (ClinicalTrials.gov NCT02355782, NCT02222545). However, complement activation seems to be a late event in TAM pathogenesis (5), and eculizumab is supposed to target only late phases of organ damage. Regarding the important role of endothelial damage in TAM (14), clear efficacy of defibrotide in clinical studies of liver VOD (23), and *in vitro* studies demonstrating damaged cultured endothelium by calcineurin inhibitors (17, 24), it appears an attractive option for TAM therapy (9–11). While the reported medium duration of defibrotide treatment in pediatric HSCT recipients with TAM is 57.5 days (11), in our patient, a short-term (7 days) course of defibrotide therapy led to full resolution of CNS injury symptoms and significant improvement of renal function. Interestingly, persistent fever of unknown origin, developing for 2 weeks before TAM, also resolved with defibrotide therapy initiation. We presume the resolution of fever was a part of the resolution of neurologic impairment, although it also supports the demonstrated *in vitro* anti-inflammatory properties of defibrotide (25).

The mechanism of defibrotide action remains unknown, but anticoagulant and fibrinolytic properties are demonstrated by modulating the release of anticoagulant and fibrinolytic factors by endothelial cells and by direct enhanced plasmin activity (26, 27). Thus, the most common adverse effect (up to 23%) of defibrotide is hemorrhage (23, 28, 29). At day 4 of defibrotide treatment, our patient developed epistaxis, followed by renal hemorrhage and hemorrhagic cystitis. No coagulopathy was demonstrated. To our knowledge, there are reports of epistaxis and hemorrhagic cystitis following defibrotide use (28, 29), but no reported data on renal hemorrhage. Owing to normal coagulation and the risks of renal thrombosis and obstruction, no pro-coagulation therapy was initiated to stop hemorrhage. At day 7 after the hemorrhage commenced, it resolved spontaneously. Importantly, the risks of hemorrhagic complications with defibrotide therapy cannot be predicted and controlled with standard coagulation investigations; hence, other diagnostic parameters should be evaluated.

Patients developing AKI and requiring dialysis after HSCT have significantly lower survival (30). The development of AKI in the early post-transplant period is significantly associated with CKD development (31). Our patient, a year after HSCT, has stage 2 CKD.

To conclude, defibrotide is an attractive rescue therapy for numerous HSCT-associated endothelial damage syndromes, where the lack of potentially curative therapies significantly narrows survival, but its use is limited by the high cost. We report successful treatment of severe TAM with a short course of defibrotide in a pediatric patient. The treatment was followed by multiple hemorrhages, which resolved spontaneously. Randomized studies in bigger cohorts of patients are needed to evaluate defibrotide safety and efficacy profiles in patients with TAM.

## Data Availability Statement

The datasets generated for this study are available on request to the corresponding author.

## Ethics Statement

The studies involving human participants were reviewed and approved by Dmitry Rogachev National Medical Center of Pediatric Hematology, Oncology and Immunology. Written informed consent was obtained from the parents for the publication of this case report.

## Author Contributions

AL was in charge of patient and wrote the manuscript. MA, IS, and IK performed renal assessment, HSCT, and intensive care, respectively. AS, DB, and AM were in charge of immunology, HSCT, and hematology care. All authors contributed to the manuscript preparation.

## Conflict of Interest

The authors declare that the research was conducted in the absence of any commercial or financial relationships that could be construed as a potential conflict of interest.
